# Clinical Efficacy of Endoscopic Surgery for Intracranial Abscesses

**DOI:** 10.7759/cureus.99492

**Published:** 2025-12-17

**Authors:** Kazuhiro Tohara, Takamitsu Iwata, Aya Ozaki, Eisaku Terada, Ryuichiro Kajikawa, Hitoshi Akazawa, Takashi Tsuzuki, Haruhiko Kishima

**Affiliations:** 1 Department of Neurosurgery, Sakai City Medical Center, Sakai, JPN; 2 Department of Otorhinolaryngology - Head and Neck Surgery, Sakai City Medical Center, Sakai, JPN; 3 Department of Neurosurgery, The University of Osaka Graduate School of Medicine, Suita, JPN

**Keywords:** brain abscess, endoscopic surgery, intraventricular rupture of brain abscess, subdural empyema, ventriculitis

## Abstract

Intracranial abscesses, including brain abscesses, ventriculitis, and subdural empyema, are critical conditions with high mortality rates. Conventional treatments involve antibiotics and surgical drainage or curettage; however, neuroendoscopic surgery has shown promising outcomes and requires further study.

Herein, we present three cases that were successfully managed using neuroendoscopic surgery. Two patients had brain abscesses with ventricular rupture secondary to dural defects and were treated with transventricular endoscopic removal and dural repair. The third patient, a male in his 90s, had subdural empyema that was treated effectively via burr hole endoscopic drainage under local anesthesia. All patients survived, highlighting the minimally invasive and effective nature of neuroendoscopic interventions.

Neuroendoscopic surgery has potential advantages in the management of intracranial abscesses by providing minimally invasive access, enhanced visualization for precise drainage, and additional diagnostic insights. These cases support the incorporation of neuroendoscopy as an effective and less invasive treatment option, especially in severely ill or elderly patients.

## Introduction

Intracranial abscesses result from various causes, including underlying conditions such as human immunodeficiency virus infection, immunosuppressant use, disruption of the brain's protective barriers through surgery or trauma, mastoiditis, or systemic infections [1]. Previous reports suggest otitis media and mastoiditis as the leading causes (32%), followed by surgical interventions (14%), cardiac disease (13%), and hematogenous infections (13%) [2]. The mortality rate ranges from 5% to 32% [3], with recent studies still indicating a high fatality rate of approximately 21% [4]. Treatments include antibiotic therapy, aspiration, or surgical excision [1,3,5,6]. Recently, endoscopic removal of brain abscesses has been reported, and its utility is under discussion [7,8]. In cases where brain abscesses develop due to adjacent infections spreading through dural defects, surgical duraplasty in addition to abscess drainage is required [9]; however, the appropriate timing and methodology remain unestablished. The prognosis is particularly poor when abscesses rupture into the ventricular system [10-12], with up to 50% mortality [13], indicating the need for effective evidence-based treatment strategies.

We previously reported a case of a brain abscess with intraventricular rupture treated via transventricular neuroendoscopic surgery [14]. While neuroendoscopic procedures are established for hydrocephalus-related conditions, such as third ventriculostomy and septostomy, they may also be beneficial for brain abscesses with ventricular involvement. Subdural empyemas can result from hematogenous spread or adjacent infections, such as sinusitis or otitis media [15,16], with sinusitis accounting for 67% of cases, followed by meningitis (10%), trauma (8%), and others [17]. Subdural empyemas also carry a poor prognosis, with mortality reported between 6% and 35% [18]. While burr hole drainage, craniotomy, or craniectomy are standard treatments [17,19], craniotomy/craniectomy has been associated with better outcomes regarding neurological improvement, mortality, and reoperation rates [17,20]. However, due to the high invasiveness of these procedures, particularly in elderly patients or those with poor general condition, endoscopic removal of subdural empyemas via burr hole has been reported as a less invasive and successful approach [7,21,22].

Herein, we present a case series comprising two patients with brain abscesses with intraventricular rupture secondary to dural defects and one patient with subdural empyema, all successfully treated with neuroendoscopic surgery. This study aimed to demonstrate the utility of endoscopic approaches for the management of intracranial abscesses.

## Case presentation

Case 1

**Figure 1:**
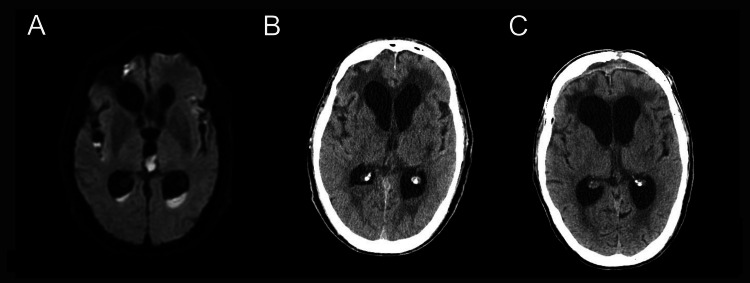
Imaging findings in Case 1 A: Brain MRI-DWI obtained prior to admission shows high signal intensity in the ventricles and subarachnoid space, consistent with intraventricular and subarachnoid abscesses. B: Head CT on admission. C: Head CT at discharge. CT, computed tomography; MRI, magnetic resonance imaging; DWI, diffusion-weighted imaging

A male in his 60s was struck by a vehicle while walking and was transferred to our hospital (Figure 1A; Figure 1B). Head computed tomography (CT) revealed a right frontal lobe contusion and anterior skull base fracture, but no cerebrospinal fluid (CSF) leakage. After three months of conservative inpatient treatment, the patient was transferred to a rehabilitation hospital with a modified Rankin Scale (mRS) score of 4. One year after the injury, the patient developed seizures, fever, and impaired consciousness. Upon admission to our hospital, the Glasgow Coma Scale (GCS) score was E1V2M4. CSF analysis showed yellowish turbid fluid with 193,141 cells/μL and a glucose level of 2 mg/dL (blood glucose, 169 mg/dL), suggesting bacterial meningitis. Magnetic resonance imaging (MRI) revealed hyperintense areas on diffusion-weighted imaging (DWI) of the ventricles and signs of sinusitis on fluid-attenuated inversion recovery (FLAIR). The patient was diagnosed with bacterial meningitis and ventriculitis. Antibiotics were administered, and ventricular drainage was initiated. On day five, a new DWI hyperintensity was noted in the anterior horn of the right lateral ventricle. On day eight, the ventricular drain was replaced under local anesthesia, and flexible neuroendoscopy was used to visualize the ventricle and remove the intraventricular abscess. A defect in the ependymal wall was exposed as a parenchymal abscess. Given the previous skull base fracture and brain contusion, we suspected a dural defect and sinus-brain communication. On day 15, dural repair was performed using a vascularized periosteal flap. Follow-up DWI revealed resolution of the abscess. On day 71, lumboperitoneal (LP) shunting was performed for postmeningitic hydrocephalus, and the patient was transferred on day 86 with an mRS score of 5 (Figure 1C). This case was previously reported by Ozaki et al. [14] from our institution, focusing on the delayed onset and postoperative course. In the present study, we reanalyzed this patient as part of our institutional case series, emphasizing the usefulness of endoscopic surgery.

Case 2

**Figure 2:**
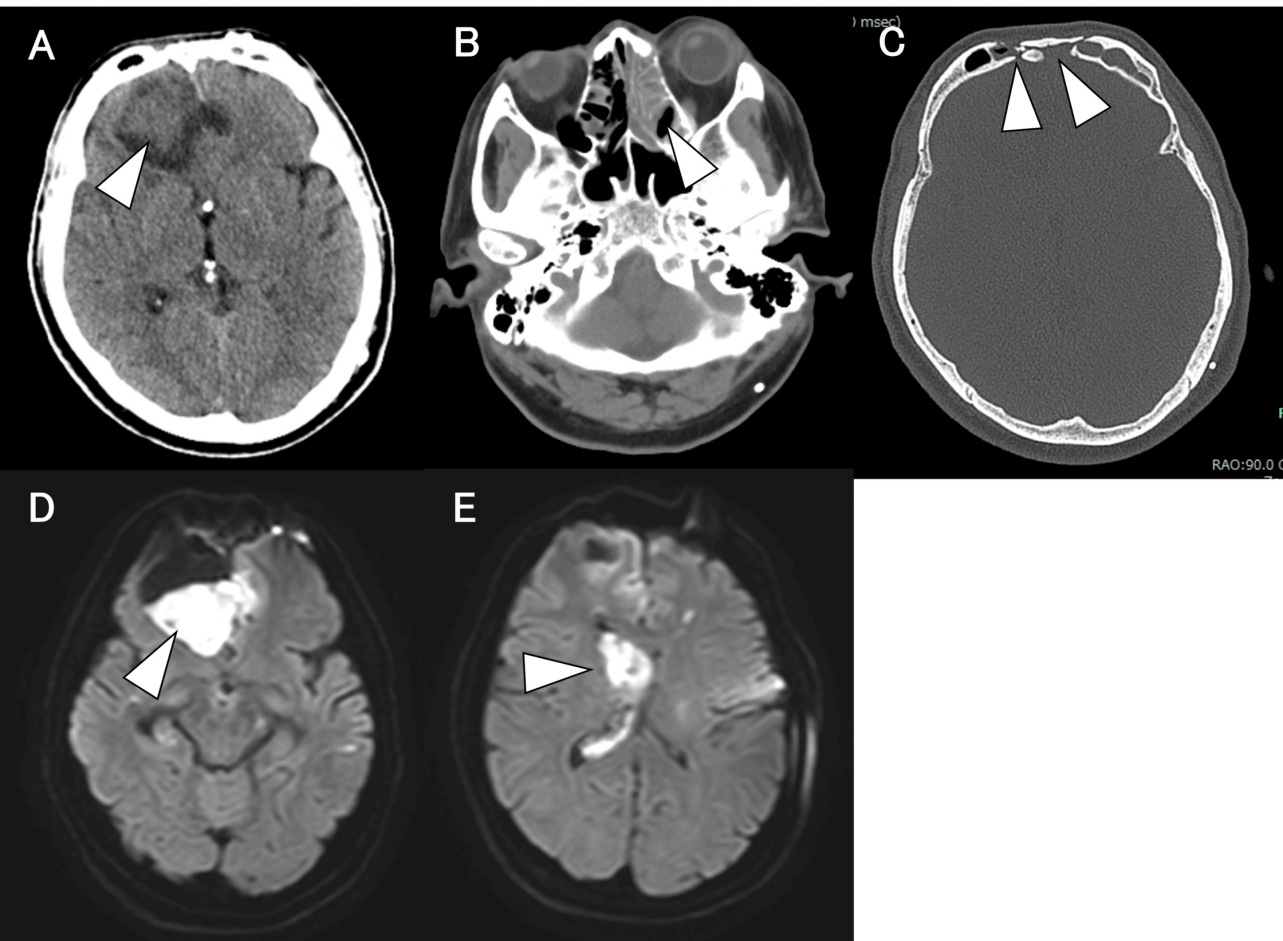
Imaging findings in Case 2 before transventricular endoscopic removal A: Head CT revealing a hypodense area with a partially hyperdense component in the right frontal lobe (arrowhead). B: Head CT showed sinusitis (arrowhead). C: Head CT confirming that the frontal sinus was patent (arrowhead). D and E: Brain MRI-DWI revealing high signal intensity areas in the right frontal lobe and right lateral ventricle, suggestive of ventriculitis and brain abscesses (arrowhead). CT, computed tomography; MRI, magnetic resonance imaging; DWI, diffusion-weighted imaging

A male patient in his 50s presented to the emergency department with fever and altered consciousness. Sixteen years prior, he had undergone clipping of a ruptured anterior communicating artery aneurysm via a basal interhemispheric approach at another institution, complicated by secondary hydrocephalus requiring the placement of a ventriculoperitoneal (VP) shunt. Upon admission, his GCS score was E4V4M5, blood pressure (BP) was 150/103 mmHg, heart rate (HR) was 131 bpm, and body temperature (BT) was 38.2°C. Laboratory studies revealed leukocytosis (21,500 cells/μL) and elevated C-reactive protein (CRP) (14.6 mg/dL). CSF analysis revealed a cell count of 188 cells/μL and a glucose level of 73 mg/dL, with a negative Gram stain and no definitive evidence of meningitis. The patient was admitted for further evaluation, and intravenous ceftriaxone (2 g/day) was initiated. On day four, CT revealed a lesion in the right frontal lobe exhibiting low-density areas with surrounding high-density areas, consistent with an abscess (Figure 2A).

**Figure 3:**
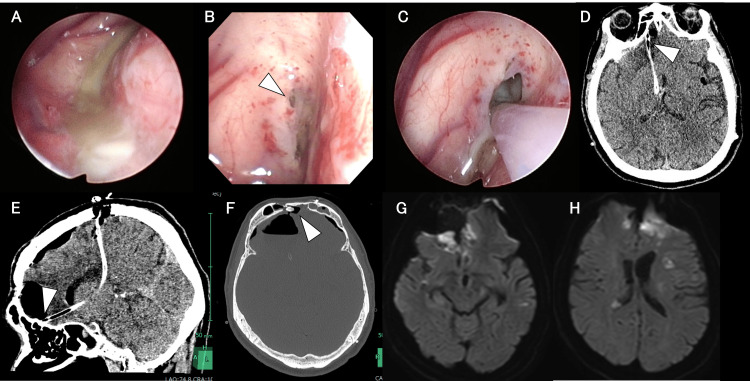
Intraoperative findings (A-C) and postoperative imaging findings (D-H) of endoscopic intraventricular removal in Case 2 A: The right lateral ventricle was filled with abscesses. B: A rupture point between the lateral ventricle and the parenchymal brain abscess was identified (arrowhead). C: A drainage catheter is inserted into the brain parenchymal abscess through the lateral ventricle. D and E: Head CT demonstrating the placement of a drainage catheter into the brain parenchymal abscess cavity through the lateral ventricle (each arrowhead). F: Head CT reveals the presence of air around the opening of the frontal sinus (arrowhead). G and H: Brain MRI-DWI confirmed that the abscesses in the right frontal lobe and right lateral ventricle were mostly removed. CT, computed tomography; MRI, magnetic resonance imaging; DWI, diffusion-weighted imaging

CT additionally demonstrated sinusitis (Figure 2B) and an open frontal sinus, suggesting a possible complication related to the previous surgery (Figure 2C). MRI-DWI on day five confirmed hyperintense lesions indicative of abscess formation in the bilateral frontal lobes and right lateral ventricle (Figure 2D and Figure 2E). The patient was diagnosed with ventriculitis and an intraparenchymal brain abscess with intraventricular rupture. Emergency surgery was performed under local anesthesia, involving the placement of a burr hole and ventricular drainage, followed by transventricular endoscopic removal of the abscess. Endoscopic inspection revealed that the purulent material completely filled the right lateral ventricle (Figure 3A).

Anterior advancement toward the frontal horn revealed a rupture site with purulent fluid entering the parenchymal brain abscess cavity (Figure 3B). The endoscope was carefully inserted beyond the rupture site into the abscess cavity, and thorough irrigation was performed. A drainage catheter was placed directly into the abscess cavity through the ventricle under endoscopic visualization (Figure 3C, Video 1).

**Video 1:** Intraoperative video of Case 2

**Figure 4:**
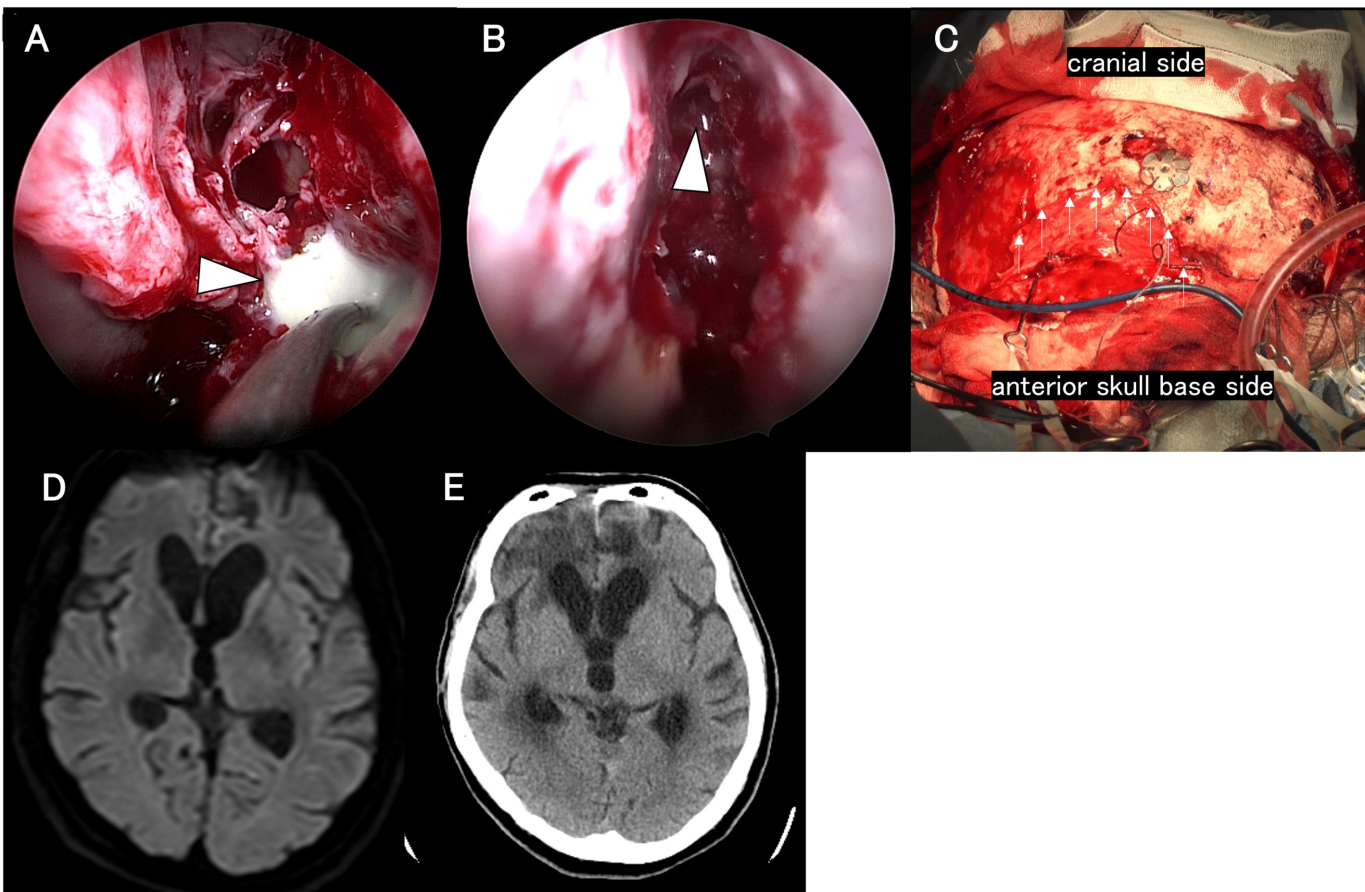
Intraoperative findings (A-C) and postoperative imaging (D, E) following endoscopic sinus surgery and CSF leak repair in Case 2 A: The abscess was observed in the left maxillary sinus (arrowhead). B: CSF leakage from the frontal sinus is confirmed (arrowhead). C: The frontal sinus opening is closed using the periosteum and fascia (arrows). D: Brain MRI-DWI on day 59 reveals no recurrence of the brain abscess. E: Head CT on day 84. CSF, cerebrospinal fluid; CT, computed tomography; MRI, magnetic resonance imaging; DWI, diffusion-weighted imaging

The VP shunt was removed, and additional ventricular drainage was established via the left frontal horn. Postoperative CT on day five confirmed appropriate positioning of the catheter through the lateral ventricle into the abscess cavity (Figure 3D and Figure 3E). Air near the frontal sinus opening suggested a communication pathway between the frontal sinus and the abscess (Figure 3F). Follow-up MRI-DWI on day seven confirmed near-complete removal of the abscess (Figure 3G and Figure 3H). Given these findings and intraoperative observations, we suspected that the open frontal sinus, resulting from prior aneurysm clipping surgery, facilitated bacterial spread from the sinus into the cerebral parenchyma and subsequently into the ventricular system. An otolaryngological consultation was requested, and endoscopic sinus surgery with septoplasty was performed on day six. Significant pus accumulation was observed intraoperatively in the left maxillary sinus, and CSF leakage was confirmed in the left frontal sinus (Figure 4A, Figure 4B, and Video 1).

Streptococcus intermedius was identified in the CSF cultures on day five, and nasal cultures on day six yielded Streptococcus intermedius and Parvimonas micra, confirming the spread from the sinus infection to the CSF. Consequently, intravenous metronidazole (1500 mg/day) was added to the ceftriaxone regimen on day seven. On day 13, the CSF leakage was surgically repaired to close the communication. Using an endoscope placed intranasally, saline infusion confirmed leakage into the frontal sinus. After removing a portion of the bone flap from the previous clipping surgery, the dural defect and frontal sinus opening were sealed with a vascularized periosteal flap and fibrin glue (Figure 4C). Intradural inspection confirmed direct communication between the abscess cavity and frontal sinus (Video 1). After repair, the surgical site was flooded with saline, and no residual leakage was confirmed endoscopically. The abscess drain on the right side was removed, and the left ventricular drain was replaced. Although CSF analysis from the ventricular drain on day 12 still showed significant pleocytosis (16,864 cells/µL), improvement to 162 cells/µL was observed by day 24. A spinal drainage catheter was placed on day 32, and the left ventricular drain was removed on day 35. The spinal drain was removed on day 45; however, a follow-up CT revealed gradual ventricular enlargement. A tap test performed on day 73 demonstrated improvement in consciousness, prompting the placement of an LP shunt. No recurrence of abscesses was noted on subsequent imaging (Figure 4D and Figure 4E). The patient was transferred to a rehabilitation hospital on day 87 with an mRS score of 5.

Case 3

**Figure 5:**
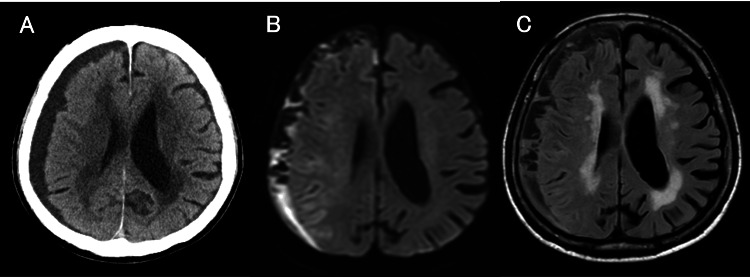
Imaging findings before endoscopic abscess removal in Case 3 A: Head CT reveals a slightly hypodense fluid collection in the right subdural space. B: Brain MRI-DWI revealed a mixture of high- and low-signal-intensity areas in the right subdural space along with a focal high-signal area on the brain surface, suggestive of a subdural abscess. C: FLAIR MRI reveals a mixture of hyperintense and hypointense areas suggestive of a pus-filled structure. CT, computed tomography; MRI, magnetic resonance imaging; DWI, diffusion-weighted imaging; FLAIR, fluid-attenuated inversion recovery

A male in his 90s presented with fever and altered consciousness (GCS E3V3M5, BP 199/99 mmHg, HR 131 bpm, BT 38.3°C). Laboratory studies showed mild leukocytosis (8,550 cells/μL, CRP 1.18 mg/dL). CSF was clear with 41 cells/μL and a glucose level of 57 mg/dL, but the film array detected Streptococcus agalactiae. Urine culture confirmed this finding, and the patient was diagnosed with a urinary tract infection and meningitis. Ampicillin was initiated at a dose of 12 g/day. Head CT on admission revealed bilateral subdural effusions that were right-dominant (Figure 5A).

**Figure 6:**
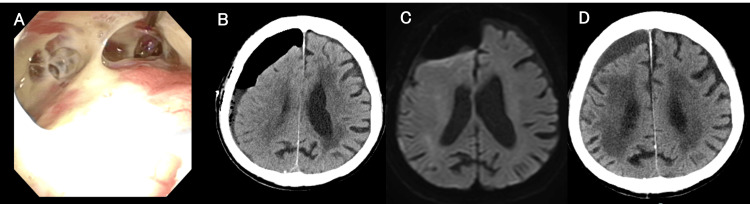
Intraoperative findings (A) and postoperative imaging findings (B-D) of endoscopic abscess removal A: A purulent collection was observed in the subdural space. B: Head CT confirmed that the subdural abscess had been largely evacuated. C: Brain MRI-DWI confirmed that the abscess had been substantially removed. D: Head CT on day 48 reveals no evidence of subdural abscess recurrence. CT, computed tomography; MRI, magnetic resonance imaging; DWI, diffusion-weighted imaging

On day nine, MRI revealed high-signal areas in the right subdural space with septation and cortical enhancement, suggesting subdural empyema (Figure 5B and Figure 5C). Emergency drainage and neuroendoscopic abscess removal via a single burr hole were performed under local anesthesia using a flexible endoscope. The purulent material was visualized and suctioned. The cavity was irrigated, and a subdural drain was placed (Figure 6A and Video 2).

**Video 2:** Endoscopic evacuation of subdural empyema

Postoperative imaging revealed successful abscess removal (Figure 6B). On day 16, DWI confirmed resolution (Figure 6C). On day 27, pneumonia developed due to Klebsiella pneumoniae, and the antibiotics were adjusted. After six weeks of antibiotic treatment, with no recurrence on imaging (Figure 6D), the patient was discharged on day 53 with an mRS score of 5.

## Discussion

We successfully treated two cases of brain abscesses with intraventricular rupture due to dural defects using transventricular endoscopic removal, and one case of subdural empyema using burr hole endoscopic drainage, with all patients surviving. Brain abscesses with intraventricular rupture are known to have a mortality rate ranging from 84% to 100% [23], and subdural empyemas have reported mortality rates between 6% and 35% [18]. Thus, survival in all our cases indicated effective management. Recently, various treatment strategies have been explored for subdural and brain abscesses and ventriculitis. Transnasal endoscopic surgery has been reported for brain abscesses and subdural empyemas originating from sinusitis [24,25]. Neuroendoscopic lavage is considered effective for the treatment of ventriculitis [26]. However, in most reported cases, the underlying cause is related to ventriculoperitoneal shunt placement or craniotomy. In contrast, Cases 1 and 2 developed ventriculitis secondary to a dural injury. Moreover, reports on neuroendoscopic treatment of subdural empyemas remain sparse [7,21,22].

The advantages of endoscopic treatment identified from our experience are as follows. First, endoscopic treatment can be combined effectively with conventional surgical methods. Ventricular drainage is a standard treatment for brain abscesses involving ventricular rupture [3]. Additionally, burr hole drainage is a common approach for subdural empyemas [19]. In our cases, endoscopic treatment was combined with ventricular drainage for brain abscesses and added to conventional drainage methods for subdural empyema. Importantly, these procedures were successfully performed under local anesthesia, demonstrating lower invasiveness than craniotomy. Notably, the elderly patient (Case three) benefited significantly from this minimally invasive approach due to the reduced anesthetic risk. Prompt intervention is recommended for subdural empyemas and ventriculitis [20,27], and our experience suggests that neuroendoscopic resection under local anesthesia is a viable emergency option.

Second, endoscopy offers enhanced visualization. Traditional aspiration or burr hole drainage methods are performed blindly. Endoscopic visualization enables precise aspiration, potentially contributing to more effective infection control. Particularly in Case 3, the internal septate structures of the abscess were directly visualized, allowing near-complete intraoperative removal. Nayan et al. demonstrated that craniotomy achieved a greater reduction in empyema volume on pre- and postoperative CT imaging compared to burr hole drainage and was associated with better neurological outcomes [20]. Compared with craniotomy, endoscopic burr hole surgery for subdural empyema is less invasive, and its superior visualization enables more complete empyema evacuation, potentially leading to improved neurological outcomes.

In Case 2, precise visualization of the rupture site enabled the placement of the drainage catheter directly into the abscess cavity. Such precise visualization also informed the subsequent dural repair strategies in Cases 1 and 2, emphasizing the utility of endoscopy for strategic treatment planning and improved outcomes. Our findings are limited to a small case series; thus, larger studies are needed. However, considering the rarity of brain abscesses with intraventricular rupture and subdural empyema, conducting extensive retrospective and prospective studies may be challenging. Therefore, continued accumulation of experience and case reports is essential.

## Conclusions

Although evidence for the treatment of brain abscesses with intraventricular rupture and subdural empyema remains limited, our three illustrative cases, two brain abscesses with intraventricular rupture and one subdural empyema, demonstrate that neuroendoscopic surgery can achieve effective infection control and survival while minimizing surgical invasiveness. In these patients, neuroendoscopy allowed abscess removal or drainage and irrigation of the infected cavity through only a small opening in the skull instead of a large craniotomy. Endoscopy may improve outcomes by providing a minimally invasive treatment, better visualization intraoperatively, and valuable intraoperative diagnostic information.
